# Autistic Traits Mediate Reductions in Social Attention in Adults with Anorexia Nervosa

**DOI:** 10.1007/s10803-020-04686-y

**Published:** 2020-09-10

**Authors:** Jess Kerr-Gaffney, Luke Mason, Emily Jones, Hannah Hayward, Amy Harrison, Declan Murphy, Kate Tchanturia

**Affiliations:** 1 Department of Psychological Medicine, Institute of Psychiatry, Psychology, and Neuroscience, King’s College London, 103 Denmark Hill, London, SE5 8AZ UK; 2 Centre for Brain & Cognitive Development, Birkbeck, University of London, London, UK; 3 Department of Forensic & Neurodevelopmental Sciences, Institute of Psychiatry, Psychology, and Neuroscience, King’s College London, London, UK; 4 Department of Psychology and Human Development, University College London, London, UK; 5 Psychological Medicine Clinical Academic Group, National Eating Disorders Service, South London and Maudsley NHS Trust, London, UK; 6 Department of Psychology, Ilia State University, Tbilisi, GA USA

**Keywords:** Anorexia nervosa, Social attention, Eye-tracking, Autism spectrum disorder, Comorbidity

## Abstract

Anorexia nervosa (AN) is associated with difficulties in social and emotional functioning. A significant proportion of individuals with AN show autistic traits, which may influence social attention. This study examined attention to faces and facial features in AN, recovered AN (REC), and healthy controls, as well as relationships with comorbid psychopathology. One hundred and forty-eight participants’ eye movements were tracked while watching a naturalistic social scene. Anxiety, depression, alexithymia, and autistic traits were assessed via self-report questionnaires. Participants with AN spent significantly less time looking at faces compared to REC and controls; patterns of attention to individual facial features did not differ across groups. Autistic traits mediated the relationship between group and time spent looking at faces.

**Electronic supplementary material:**

The online version of this article (10.1007/s10803-020-04686-y) contains supplementary material, which is available to authorized users.

Anorexia nervosa (AN) is a severe psychiatric disorder characterised by an intense fear of gaining weight, persistent behaviour to restrict energy intake, and disturbances in the way one’s body or shape is experienced (American Psychiatric Association [APA] 2013). AN is associated with high levels of psychiatric comorbidity. For example, recent research suggests that between 4 and 52.5% of individuals with AN show clinically significant levels of autism spectrum disorder (ASD) traits, scoring above clinical cut-offs on assessment tools such as the Autism Diagnostic Observation Schedule, 2nd edition (ADOS-2) (Westwood et al. 2018; Westwood and Tchanturia 2017). The presence of ASD traits in individuals with AN has been associated with more frequent and longer inpatient stays (Nazar et al. 2018), poorer outcomes (Anckarsäter et al. 2012; Nielsen et al. 2015; Wentz et al. 2009), and less improvement during treatment (Stewart et al. 2017; Tchanturia et al. 2016). While studies investigating the prevalence of AN in individuals with ASD are currently lacking, there is evidence to suggest those with ASD show significantly more eating disorder symptoms than non-autistic people (Nickel et al. 2019). Greater autistic traits in childhood are associated with a greater risk of disordered eating in adolescence (Solmi et al. 2020), and 27% of women with ASD report clinically significant levels of eating disorder symptoms (Kalyva 2009; Spek et al. 2019). In addition, adults with ASD are more likely to be in non-healthy weight categories (underweight, overweight, or obese) than non-autistic people (Sedgewick et al. 2020).

Contemporary models of AN emphasise interpersonal difficulties as key factors in the development and maintenance of the disorder (Fairburn et al. 2003; Treasure and Schmidt 2013). During the illness, individuals with AN show high levels of social anhedonia (reduced interest or pleasure from social situations) and social anxiety, difficulties that persist after recovery (Harrison et al. 2014; Kerr-Gaffney et al. 2018; Tchanturia et al. 2012). Individuals with AN also report poorer social skills, and use fewer positive social problem-solving strategies compared to healthy controls (HCs) (Rhind et al. 2014; Sternheim et al. 2012). Further, research has documented lower levels of social support, reduced social networks, and high levels of isolation in those with AN (Adenzato et al. 2012; Arkell and Robinson 2008; Doris et al. 2014; Gillberg et al. 1994; Tiller et al. 1997). Importantly, there is evidence to suggest that social difficulties may be present before illness onset. Up to two thirds of individuals with AN report having early social difficulties, and are more likely to report having no childhood friends and a history of being bullied than their unaffected peers (Cardi et al. 2018a; Fairburn et al. 1999; Gillberg and Råstam 1992; Lie et al. 2019). Given that interpersonal problems are associated with poorer outcomes in those with AN (Franko et al. 2013; Zipfel et al. 2000), it is important to understand possible underlying cognitive mechanisms.

Attending to others’ eye gaze, facial expressions, posture, and gestures is key to effective social interaction, as these non-verbal cues convey important information about an individual’s emotions, thoughts, and intentions. In typical human development, social information is highly salient, with infants as young as a few days old showing a preference for face stimuli (Reynolds and Roth 2018). Indeed, reductions in social attention are among the first characteristics of disorders of social communication such as ASD (Jones et al. 2014). Reduced attention to faces, and particularly the eyes, has been found to predict degree of social impairment and emotion recognition in those with ASD (Corden et al. 2008; Falkmer et al. 2011; Klin et al. 2002; Müller et al. 2016). Differences in social attention have also been found in individuals with social anxiety disorder, where avoidance of eye contact and other social gestures are hypothesised to act as safety behaviours. Such behaviours are an attempt to reduce anxiety or prevent a feared negative event, but perpetuate anxiety in the long term. As a result of this reduction in attention to social information, fears around social evaluation are prevented from being disconfirmed, and positive social experiences are not registered, thereby maintaining the disorder (Chen and Clarke 2017).

It is possible then that differences in social attention might relate to interpersonal difficulties associated with AN. However, much less research has examined social attention in this population. Several studies have used the pictorial Stroop or dot-probe task, reporting an attentional bias towards angry and rejecting faces and away from neutral and compassionate facial expressions in individuals with AN (Cardi et al. 2012, 2014; Harrison et al. 2010). However, these reaction time (RT) based paradigms have a number of limitations. For example, increased RTs to faces in the emotional Stroop task are interpreted as increased attention, as the emotional salience of the face interferes with one’s response latency. However, it is equally possible that participants diverted their attention away from the stimulus, thereby increasing RTs (Aspen et al. 2013). Further, these tasks use isolated static faces, and are therefore unable to provide much insight into attention to social information in real life, dynamic environments, as well as potential differences in attention to facial features.

A few studies have therefore used eye-tracking paradigms to directly capture attention to social stimuli in AN. For example, Pinhas et al. (2014) found that participants with AN payed less attention to images of social interactions when presented alongside images of body shapes, whereas HCs spent similar amounts of time looking at both types of image. Similarly, Watson et al. (2010) found that a small group of weight-restored AN spent less time looking at faces when the body was also present within the image, compared to HCs. Importantly, when faces were presented alone, participants with AN looked significantly less at the eyes than HCs, providing the first eye-tracking evidence of abnormal social attention in the absence of disorder-relevant body stimuli. This finding was replicated in a study by Harrison et al. (2019), who found reduced attention to the eyes of both static face images, videos of social interactions, and during a real-life social interaction in acute AN compared to recovered AN and HCs. Similar to findings in ASD, reduced attention to the eyes was associated with greater self-reported social difficulties. In summary, there is preliminary evidence to support reduced attention to social information, and particularly the eyes, in individuals with AN.

Thus far, studies using dynamic stimuli have not measured attention to other parts of the face, such as the nose and mouth, in individuals with AN. Although areas such as the nose may convey less information about emotions or other mental states, eye-tracking research has shown that healthy individuals generally shift between looking at the eyes, nose, and mouth during both free-viewing and face recognition tasks (Hsiao and Cottrell 2008; Vo et al. 2012; Walker-Smith et al. 1977). In addition, research in individuals with ASD has shown increased attention to the mouth is associated with better social adjustment, suggesting that those with ASD may employ compensatory strategies to derive meaning from other parts of the face, differently from HCs (Klin et al. 2002). The findings from ASD are particularly important for the study of social attention in individuals with AN, given the emerging literature documenting comorbidity between the two conditions (Gillberg 1983; Westwood et al. 2018).

Thus, the primary aim of the current study was to examine attention to faces, as well as core facial features (eyes, nose, mouth), while viewing naturalistic, dynamic social scenes in individuals with AN, recovered AN, and HCs. It was hypothesised that individuals with acute AN would spend less time looking at faces and eyes of faces compared to HCs. We predicted intermediate levels of attention in those recovered from AN. A second aim was to examine whether ASD traits were associated with social attention. Further, given that AN is also associated with high levels of depression (Godart et al. 2015), anxiety (Kerr-Gaffney et al. 2018; Swinbourne and Touyz 2007), and alexithymia (Westwood et al. 2017a), factors which may themselves alter socio-cognitive processes (Claudino et al. 2019; Duque and Vázquez 2015; Frazier et al. 2017; Fujiwara 2018; Gregory et al. 2019), a third aim was to examine whether comorbid psychopathology was associated with social attention. It was hypothesised that high levels of ASD traits would be associated with less time spent looking at faces and eyes, as well as a longer delay until first fixation on the face.

## Methods

### Participants

Ethical approval was obtained from the National Health Service (NHS) Research Ethics Committee (Camberwell St Giles, 17/LO/1960). All participants were required to be between 18 and 55 years old and fluent in English. Exclusion criteria were a history of brain trauma or learning disability. HC participants were recruited through a King’s College London email circular and posters around campuses. Before taking part, HC participants were screened using the Structured Clinical Interview for DSM-5 Disorders, research version (SCID-5-RV; First et al. 2015), to ensure they did not show symptoms consistent with any psychiatric disorders. In addition, HCs were required to have a body mass index (BMI) between 19 and 27.

In addition to the university advertisements, participants with current or past AN were recruited through online advertisements (B-eat, call for participants, MQ mental health) and through two specialist eating disorder services in London. Participants were interviewed with the SCID-5-RV to confirm a current or past diagnosis of AN. Participants with AN were required to have a BMI ≤ 18.5, and recovered participants a BMI between 19 and 27.

### Materials

The eye-tracking stimulus material was a movie clip from the Dynamic Images and Eye Movements database (“Fifty People One Question: Brooklyn”, https://thediemproject.wordpress.com/), in which several pedestrians in Brooklyn, New York are interviewed and are seen speaking to the camera. The original audio accompanying the clip was replaced with background music, in order to control for the effects of speech comprehension on attention (Vo et al. 2012). The clip was chosen for its depiction of what would typically be seen when engaging in a natural social interaction with one or two people, whereby actors began and stopped talking, made and broke eye contact with their partner, and looked towards the camera. The clip was also chosen for its lack of body information (people were seen from the shoulders up), as this is known to be a salient class of stimuli in individuals with AN (Pinhas et al. 2014). The clip lasted 42 s, and participants were asked to simply view the clip as they would watch television. Total looking times (in seconds) to the screen were computed to control for overall attention to the stimulus, and total fixation duration to each area of interest (AOI) was calculated (as a proportion of total valid samples).

**Fig. 1:**
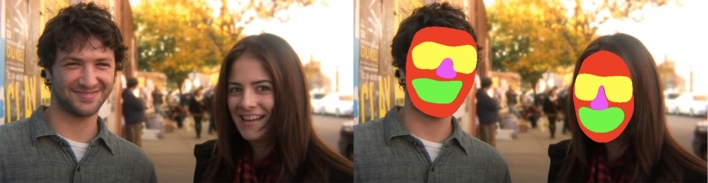
A single frame from the video clip “Fifty People, One Question: Brooklyn” (left), and with the areas of interest (AOIs) overlaid (right)

Figure 1 depicts a single frame from the video, depicting the AOIs of interest (whole face, eyes, nose, and mouth). Note that the face AOI included the core features as well as the outer regions of the face. AOIs were drawn on each individual frame of the video using Apple Motion (Apple 2019). Core feature AOIs encompassed the features, but also extended outward to include emotionally expressive regions that border the features themselves (e.g., the eye region includes the eyebrows). Face AOIs followed the outline of the face, starting just below the hairline and following along the jawline.

To capture attention to faces, two dependent measures were used: fixation duration to face AOIs, and time to first fixation on the face. To capture attention to core facial features, the following dependent measures were used: fixation duration to eye, nose, and mouth AOIs, and eye-to-mouth viewing ratio, defined as fixation duration on eyes/(fixation duration on eyes + fixation duration on mouth).

The Wechsler Abbreviated Scale of Intelligence-Second Edition (WASI-II; Wechsler 2011) was used to measure IQ, to ensure groups were matched in this respect. The two subtest version was used (vocabulary and matrix reasoning).

#### Self-report Questionnaires

The Eating Disorder Examination Questionnaire (EDE-Q; Fairburn and Beglin 1994) measures severity of eating disorder symptoms, with higher scores indicating more severe psychopathology. The EDE-Q consists of 28 items, 22 of which are rated on a 7-point Likert scale. Total scores (range: 0–6) are calculated by averaging responses across these items. The scale also includes six items assessing frequency of various eating disorder behaviours, but responses can take on any value and are not included in total score calculations. HCs with a score of ≥ 2.7 were excluded from analyses to ensure those with sub-threshold eating disorder symptoms were not included (Lang et al. 2016). Cronbach’s alpha was 0.93.

The Hospital Anxiety and Depression Scale (HADS; Zigmond and Snaith 1983) is a 14 item questionnaire with two subscales measuring severity of anxiety and depression. Subscale scores are interpreted as: normal (0–7), mild (8–10), moderate (11–14), and severe (15–21). Cronbach’s alpha was 0.93.

The Liebowitz Social Anxiety Scale (LSAS; Liebowitz 1987) is a 48 item questionnaire assessing severity of social anxiety symptoms. Total scores range from 0 to 144, and a score of 60 or more has been established as a cut-off indicative of social anxiety disorder (Rytwinski et al. 2009). Cronbach’s alpha was 0.97.

The twenty-item Toronto Alexithymia Scale (TAS-20; Bagby et al. 1994) measures severity of alexithymia, with three subscales: difficulty identifying feelings, difficulty describing feelings, and externally oriented thinking. Total scores range from 0 to 100, and cut-offs are as follows: ≤ 51 = no alexithymia; 52–60 = borderline alexithymia; and ≥ 61 = alexithymia (Parker et al. 1993). Cronbach’s alpha was 0.91.

The Social Responsiveness Scale-2nd Edition, adult self-report form (SRS-2; Constantino and Gruber 2012) includes five subscales, measuring symptoms associated with ASD: social awareness, social cognition, social communication, social motivation, and restricted and repetitive interests. Total scores can also be converted into *T*-scores, which are interpreted as follows: ≤ 59*T*, “normal”; 60–65*T*, “mild”; 66–75*T*, “moderate”; ≥ 76*T* “severe” range. SRS-2 scores have been shown to be associated with functional impairment in individuals with AN, however scores are independent from indicators of malnutrition such as BMI and illness duration (Kerr-Gaffney et al. 2020b). Cronbach’s alpha was 0.96.

The Work and Social Adjustment Scale (WSAS; Mundt et al. 2002) is a brief measure of functional impairment in five domains: work, home management, social leisure, private leisure, and ability to form and maintain close relationships. Scores range from 0 to 40, with a score of 20 or more indicating clinical significance. Cronbach’s alpha was 0.92.

### Procedure

Participants attended a testing session at the Institute of Psychiatry, Psychology & Neuroscience. After written informed consent was obtained, participants viewed the video clip while their eye movements were recorded using a Tobii TX300 eye-tracker. The desktop mounted eye-tracker had a sampling rate of 300 Hz, a screen resolution of 1920 × 1080, and a diagonal screen size of 23″. During tracking, infrared diodes generate reflections on the participant’s retinas and corneas. From this reflection, the angular rotation of each eye is estimated. Before stimulus presentation, a five-point calibration procedure was run. Calibration relates the angular rotation of each eye to the corresponding x and y coordinates on the screen surface. Participants were seated approximately 60 cm from the screen. Stimulus presentation, behavioural data, and eye-tracking data were managed and recorded using custom-written Matlab software (Task engine 2015, https://sites.google.com/site/taskenginedoc).

After eye-tracking, the first author administered the WASI-II and the participant completed the questionnaires. Weight and height measurements were taken to calculate BMI (weight/height^2^). Participants were reimbursed £20 for their time.

### Analysis

Histograms and Q-Q plots were inspected to check for normal distributions. Where variables were positively skewed (as was the case for age and time to first fixation to the face), a logarithmic transformation was applied for analyses, however original values (M and SD) are reported for clarity. Homogeneity of variances were assessed using Levene’s test. Group differences in psychopathology, demographic information, and attention outcome measures were assessed using one-way ANOVAs (or Welch’s ANOVA with Games-Howell post-hoc tests where the assumption of homogeneity was violated), with the exception of attention to core feature AOIs, which was assessed using a mixed ANOVA. Independent samples t-tests were used when assessing group differences between AN and REC only, and to test for differences between medicated and unmedicated participants. Chi-squared tests of homogeneity (or Fisher’s exact test where the sample size assumption was not met) were conducted for dichotomous variables.

To examine associations between psychopathology and attention, Spearman’s correlations between attention variables, psychopathology, and selected demographic variables were run. Where significant correlations were found, variables were entered into hierarchical linear regressions to determine whether dimensions of psychopathology predicted social attention, over and above group membership. Assumptions for hierarchical regressions were assessed with partial regression plots, and plots of studentized residuals against predicted values. Independence of residuals was assessed using the Durbin-Watson statistic, and outliers with studentised deleted residuals greater than ± 3 were excluded from analyses.

The SPSS macro PROCESS (Hayes 2017) was used for mediation analyses. PROCESS generates bias-corrected 95% bootstrap confidence intervals (CIs) for the indirect effect (based on 5000 samples), and is more powerful than the causal steps approach to mediation (Hayes and Preacher 2014).

## Results

### Demographics

In total, 148 participants were recruited (46 AN, 51 REC, 51 HC). Five HCs were subsequently excluded based on their EDE-Q scores and one REC participant was excluded due to a BMI > 27. A further 13 participants were excluded from analyses due to: eye-tracking equipment failure on the day of testing (n = 2), low quality eye-tracking data, defined as a proportion of valid samples of less than 0.25 (n = 9), outliers identified with residuals more than 3 SDs from the mean (n = 2). Thus, data from 129 participants were included in analyses, demographic and clinical information for which is presented in Table 1. Groups were similar in age, sex, years of education received, and IQ. Participants in the REC group had been recovered for an average of 4.44 years (SD = 4.46).

**Table 1:** Mean (SD) demographic information and psychopathology scores

	AN (N = 41)	REC (N = 48)	HC (N = 40)	Test statistics	*p* value	ηp^2^/*d*
Age (years)^a^	26.66 (8.59)	26.10 (8.15)	23.90 (4.75)	F(2,80.76) = 1.63	0.203	0.02
% female	90.2	97.9	95	Fisher’s exact test = 2.39	0.295	
BMI	15.81 (1.37)^a^	21.18 (1.91)^b^	21.75 (1.96)^b^	F(2,125) = 142.10	**< .001**	0.7
Years of education	16.21 (3.10)	16.58 (2.62)	16.42 (2.41)	F(2,125) = 0.20	0.822	0
IQ	110.00 (12.21)	110.44 (10.75)	113.46 (7.05)	F(2,80.09) = 1.88	0.159	0.02
Age diagnosed^a^	19.78 (6.77)^a^	16.36 (3.59)^b^	–	t(59.53) = 2.89	**0.005**	0.63
Illness length (years)	6.87 (7.80)	5.48 (5.69)	–	t(72.65) = 0.94	0.353	0.20
% on psychiatric medication	48.8	31.3	–	X^2^ = 2.85	0.091	
EDE-Q	3.79 (1.27)^a^	1.82 (1.53)^b^	0.50 (0.46)^c^	F(2,67.14) = 125.46	**< .001**	0.55
HADS-A	13.24 (4.53)^a^	10.83 (5.16)^a^	4.73 (2.82)^b^	F(2,80.33) = 61.11	** < .001**	0.40
HADS-D	9.46 (4.19)^a^	5.02 (4.06)^b^	1.50 (1.72)^c^	F(2,73.40) = 69.21	**< .001**	0.45
LSAS	69.18 (28.53)^a^	57.17 (30.29)^a^	27.00 (17.80)^b^	F(2,79.65) = 37.92	**< .001**	0.30
SRS-2	79.98 (30.97)^a^	70.19 (32.30)^a^	36.45 (17.26)^b^	F(2,77.72) = 39.03	**< .001**	0.30
TAS-20	57.75 (13.76)^a^	49.81 (15.08)^b^	36.00 (9.97)^c^	F(2,81.26) = 35.21	**< .001**	0.31
WSAS	21.93 (8.62)^a^	11.27 (8.74)^b^	2.75 (5.20)^c^	F(2,80.42) = 75.83	**< .001**	0.50

*AN* anorexia nervosa, *BMI* body mass index, *EDE-Q* eating disorder examination questionnaire, *HADS-A* hospital anxiety and depression scale, anxiety subscale, *HADS-D* hospital anxiety and depression scale, depression subscale, *HC* healthy control, *IQ* intelligence quotient, *LSAS* Liebowitz social anxiety scale, *REC* recovered anorexia nervosa, *SD* standard deviation, *SRS-2* social responsiveness scale, 2nd edition, *TAS-20* twenty-item Toronto alexithymia scale

Different superscripts indicate significant differences between groups, significant *p*-values are highlighted in bold

^a^Variable was log transformed for analyses, original values are displayed

**Fig. 2:**
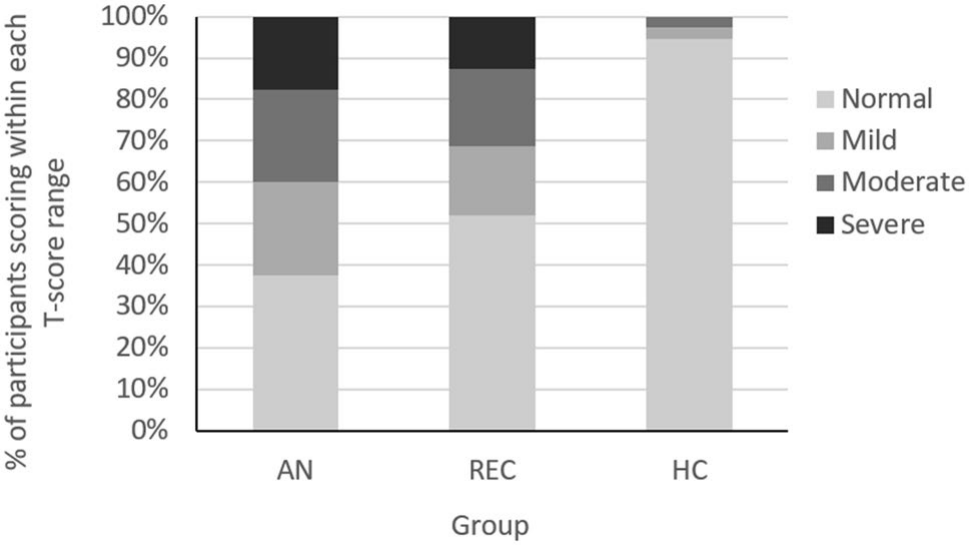
Proportion of participants scoring within each T-score range on the social responsiveness scale, adults self-report version (SRS-2). *AN* anorexia nervosa, *HC* healthy control; *REC* recovered anorexia nervosa

The distribution of SRS-2T-scores is displayed in Fig. 2. Ninety-five percent of HCs scored within the “normal” range, compared to 37.5% of participants with AN and 52.1% of REC participants. Of the HCs, 2.6% scored within the “mild” range, compared to 22.5% of participants with AN and 16.7& of REC. Similarly, 2.6% of HCs scored within the “moderate” range, compared to 22.5% of participants with AN and 18.8% of REC. Finally, 17.5% of participants with AN and 12.5% of REC scored within the “severe” range, while no HCs did.

### Attention to Faces

**Fig. 3:**
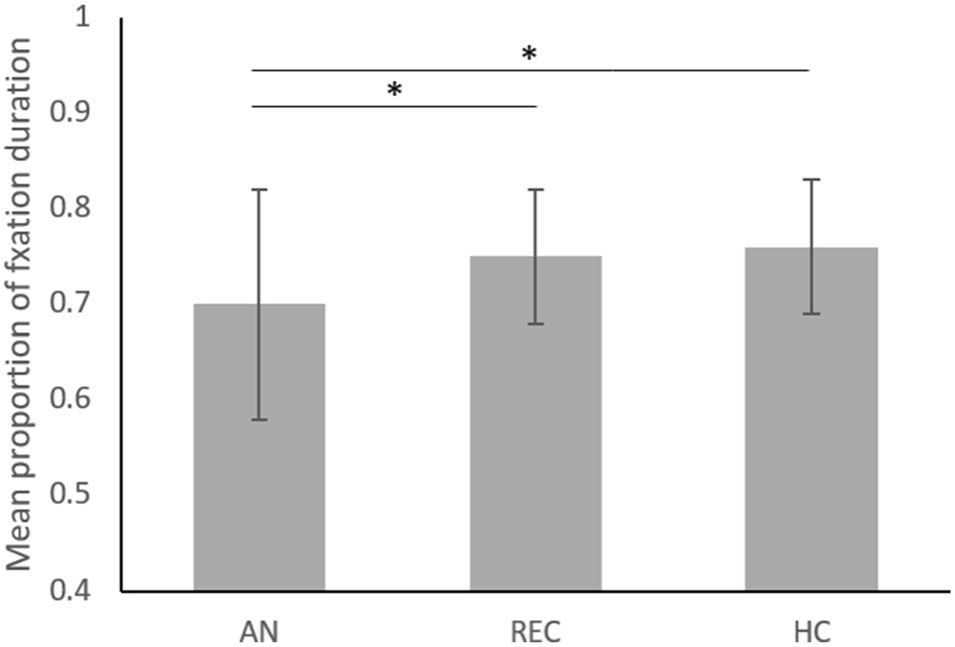
Mean proportion of time spent looking at face AOIs across groups. Error bars represent standard deviation. Significant *p*-values indicating group differences are marked with an asterisk, * < .05. *AN* anorexia nervosa, *HC* healthy control, *REC* recovered anorexia nervosa

There were no significant differences between groups in time spent looking at the screen, X^2^(2) = 1.08, p = 0.581, indicating the groups did not differ in overall attention to the stimulus. A one-way ANOVA indicated a significant difference in time spent looking at faces, F(2, 126) = 4.17, p = 0.018, ηp^2^ = 0.06. Post-hoc analyses indicated that individuals with AN looked at faces significantly less (M = 0.70, SD = 0.12) than REC (M = 0.75, SD = 0.07), p = 0.050 (95% CI, -0.09, -0.01), and HCs (M = 0.76, SD = 0.07), p = 0.025 (95% CI, − 0.10, − 0.01), as shown in Fig. 3. Regarding time to first fixation, a one-way ANOVA indicated there were no significant differences between individuals with AN (M = 0.81 s, SD = 0.50), REC (M = 0.76 s, SD = 0.28 s), or HCs (M = 0.88 s, SD = 0.68 s), F(2, 126) = 0.21, p = 0.81, ηp^2^ = 0.00.

### Attention to Facial Features

**Fig. 4:**
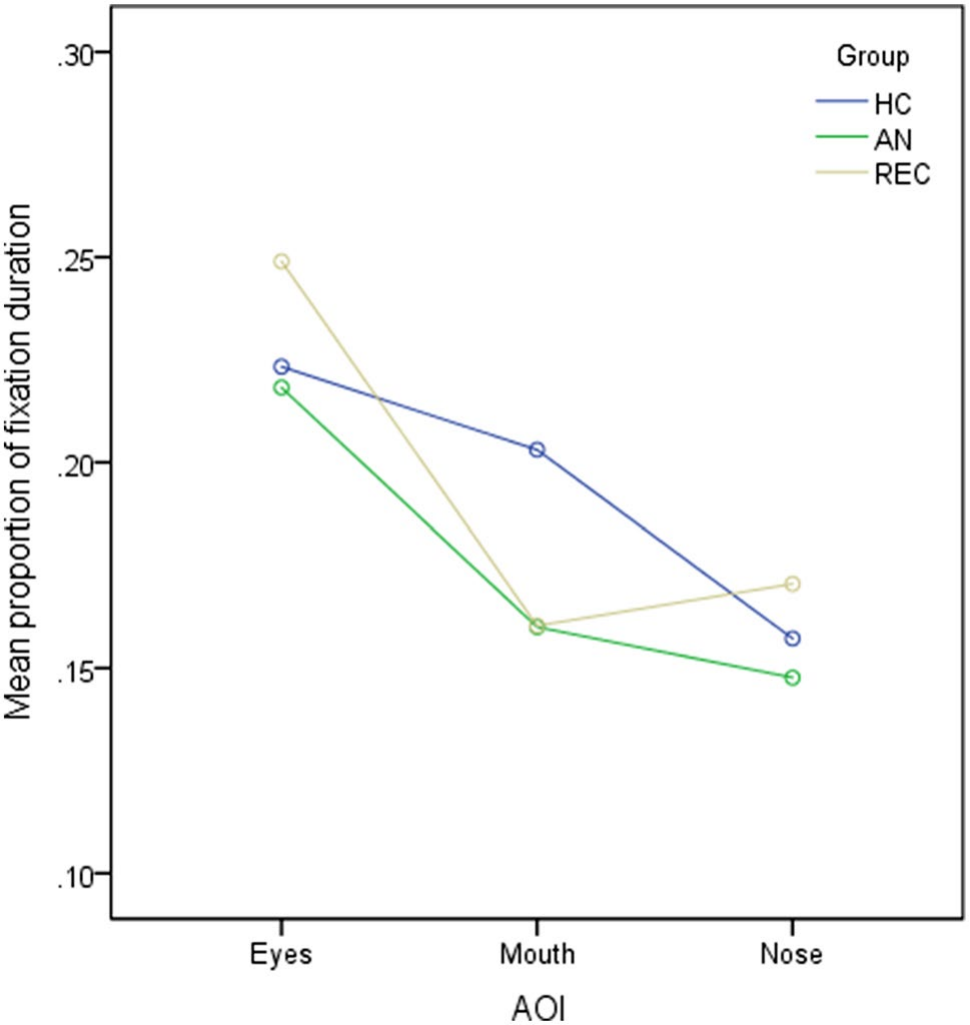
Mean proportion of time spent looking at core facial features across groups. *AOI* area of interest, *AN* anorexia nervosa, *HC* healthy control, *REC* recovered anorexia nervosa

To examine whether patterns of attention to the different facial features differed between groups, a mixed ANOVA with AOI as the within-subjects factor (eyes, mouth, and nose) and group as the between-subjects factor was run. A Greenhouse–Geisser correction was applied due to violation of Mauchly’s test of sphericity. The interaction between group and AOI was not significant, F(2.86, 180.13) = 0.96, p = 0.41, ηp^2^ = 0.02. The main effect of AOI showed a significant difference in the proportion of time spent looking at the different facial features, F(1.43, 180.13) = 11.98, p < 0.001, ηp^2^ = 0.09. Participants spent significantly more time looking at the eyes than the mouth (p = 0.016, 95% CI 0.01, 0.10) and nose (p < 0.001, 95% CI, 0.04, 0.10), as shown in Fig. 4. The main effect of group was also significant, F(2, 126) = 6.47, p = 0.002, ηp^2^ = 0.09. Post-hoc tests indicated that individuals with AN looked at AOIs less than REC (p = 0.007, 95% CI − 0.03, − 0.01) and HCs (p = 0.005, 95% CI − 0.03, − 0.01).

Further, a one-way ANOVA indicated there were no significant differences in eye-to-mouth viewing ratio between individuals with AN (M = 0.59, SD = 0.26), REC (M = 0.61, SD = 0.25), and HC (M = 0.53, SD = 0.27), F(2, 126) = 1.25, p = 0.289, ηp^2^ = 0.02.

### Relationships Between Social Attention and Psychopathology

Spearman’s correlations between attention variables (time spent looking at faces and core facial feature AOIs, time to first fixation to the face, eye-to-mouth viewing ratio), age, BMI, psychopathology scores (EDE-Q, HADS anxiety, HADS depression, TAS-20, LSAS, SRS-2), and functional impairment (WSAS scores) were run separately for each group. Given that significant correlations would be followed up with regression analyses, they were treated as exploratory and not corrected for multiple comparisons. The full table of correlations can be found in supplementary file 1. In individuals with AN, time spent looking at faces was significantly negatively correlated with TAS-20 (r = − 0.33, p = 0.040) and SRS-2 scores (r = − 0.40, p = 0.011), while time to first fixation to the face was significantly positively correlated with WSAS (r = 0.37, p = 0.017), depression (r = 0.43, p = 0.006), and anxiety scores (r = 0.34, p = 0.031). No significant correlations were found in HC or REC groups.

Given the association between SRS-2 and TAS-20 scores and time spent looking at faces in individuals with AN, a hierarchical regression was run to examine whether SRS-2 and TAS-20 scores predicted time spent looking at faces, above the effects of group. Details of each regression model are presented in Table 2. The final model (model 3) was significant, R^2^ = 0.10, F(4, 123) = 3.46, p = 0.010, adjusted R^2^ = 0.07. The addition of SRS-2 scores led to a significant increase in R^2^ (model 3), however the addition of TAS-20 scores did not (model 2). In the final model, only SRS-2 scores made a significant unique contribution to explaining the variance in time spent looking at faces. This suggests that ASD symptoms predicted looking duration to faces over and above diagnosis, with higher SRS-2 scores being associated with less attention to faces.

**Table 2:** Hierarchical regression analysis predicting time spent looking at faces from associated psychopathology scores

	Model 1	Model 2	Model 3
Group			
AN vs HC	− .27*	− .19	− .14
REC vs HC	− .04	.01	.07
TAS-20		− .12	.06
SRS-2			− .28*
R^2^	.06	.07	.10

Figures shown are standardized coefficients. Group was represented as two dummy variables

*p < .05

To further explore a possible mediational effect of SRS-2 scores, a mediation analysis was run with group as the independent variable (using indicator cording, with HCs as the reference group), SRS-2 scores as the mediator, and time spent looking at faces as the dependent variable. Bias-corrected bootstrapped CIs for the indirect effects were entirely below zero (*b*_*1*_ = − 0.03 [− 0.05 − 0.01], *b*_2_ = 0.02 [− 0.04 − 0.01]), indicating there was a significant mediation effect of group on time spent looking at faces through SRS-2 scores. The direct effect of group was not significant (*c*_1_ = − 0.02, *c*_2_ = 0.02, *p* = 0.117), indicating that group did not influence time spent looking at faces independent of its effect on SRS-2 scores. Thus, there was evidence of full mediation.

A hierarchical regression was run to investigate whether anxiety and depression predicted time to first fixation on the face, over and above the effects of group membership. Despite the significant correlation between WSAS scores and time to first fixation, this variable was not entered in the regression due to the assumed direction of causality. Results of each regression model are presented in Table 3. The full model was not significant, R^2^ = 0.02 F(4,124) = 0.57, p = 0.688, adjusted R^2^ = -0.01. None of the included variables explained significant variance in time to first fixation on the face.

**Table 3:** Hierarchical regression analysis predicting time to first fixation on the face from associated psychopathology scores

	Model 1	Model 2	Model 3
Group			
AN vs HC	− .05	− .02	− .09
REC vs HC	− .07	− .05	− .06
HADS-A		− .04	− .15
HADS-D			.20
R^2^	.00	.00	.02

Figures shown are standardized coefficients. Group was represented as two dummy variables

*p < .05

### Medication Status and Social Attention

Independent samples t-tests comparing AN and REC participants who were taking any type of psychiatric medication (n = 37) to those who were not (n = 54) indicated no significant differences in time spent looking at faces (t(44.47) = 1.24, p = 0.283), time to first fixation (t(89) = − 1.48, p = 0.142), or eye-to-mouth viewing ratio (t(89) = 0.37, p = 0.714). A mixed ANOVA with AOI as the within-subjects factor (eyes, nose, mouth) and medication status as the between-subjects factor showed that only the main effect of AOI was significant, F(1.43, 127.33) = 10.77, p < 0.001, ηp^2^ = 0.11, confirming that participants looked at the eyes more than the nose (p < 0.001, 95% CI, 0.04, 0.11) and mouth (p = 0.008, 95% CI, 0.02, 0.13). The interaction effect (F(1.43,127.33) = 0.43, p = 0.583, ηp^2^ = 0.01) and the main effect of medication status were not significant (F(1,89) = 0.49, p = 0.486, ηp^2^ = 0.01).

## Discussion

The current study aimed to examine attention to faces and core facial features in individuals with AN, REC, and HCs, using dynamic social stimuli. Given the high levels of comorbid psychopathology that often accompany AN, a second aim was to explore associations between comorbid traits and social attention. The main finding to emerge from the study was that participants with AN spent significantly less time looking at faces compared to REC and HCs. The association between group and time spent looking at faces was found to be fully mediated by ASD traits (SRS-2 scores). Contrary to our hypotheses, groups did not differ in their patterns of attention to the individual facial features, although individuals with AN spent less time overall looking at facial features overall compared to REC and HCs. Further, no group differences were found in delay to first fixation to the face. Each of these findings will be discussed in turn.

Replicating previous studies using static stimuli, our results suggest reduced attention to faces in the acute state of AN (Watson et al. 2010). Given the lack of group differences in delay to first fixation on the face, this finding suggests that individuals with AN may show initial interest in orienting to social stimuli, but disengage from such stimuli more quickly than HCs. A similar pattern of results was reported by Ketelaars et al. (2017) in women with ASD. Regarding attention to the core facial features, our results differ from those reported by Harrison et al. (2019), who found reduced attention to the eyes in both acute and recovered AN compared to HCs. However, using static face stimuli, Dinkler et al. (2019) found that those recovered from AN showed no differences in attention to eyes or mouths compared to HCs, similar to the results of the current study. Again, our results in acute AN are similar to those of Ketelaars et al. who found that women with ASD fixated all parts of the face less than HCs. These results are in contrast to a wide literature documenting reduced attention to eyes specifically in individuals with ASD (Tanaka and Sung 2016). Ketelaars and colleagues suggest their findings might be due to differences in the male and female phenotype of ASD, with females showing enhanced social motivation and better social communication than males with ASD (Harrop et al. 2018). Further, age and level of functioning may influence whether differences in social attention are found in individuals with ASD. In contrast to studies in children, adults with ASD are more likely to have undergone various social skill interventions, and as a result may be more likely to understand the importance of eye contact (Zamzow et al. 2014). Thus far, social attention research in AN has focussed exclusively on adults, however it would be of interest to measure attention to faces and facial features in children and adolescents with AN. Indeed, there is evidence to suggest that some socio-emotional difficulties may be more severe in adolescents with AN compared to adults (Lang et al. 2015).

Results of our mediation analysis suggested that reduced attention to faces in AN appears to be a result of the high levels of ASD traits present in this group. These findings are in accordance with research investigating other socio-cognitive domains in AN, where it has been demonstrated that high ASD traits predicted empathic abilities over and above AN status (Kerr-Gaffney et al. 2020a). Although the mediation effect found provides some explanation as to why social attention may be altered in individuals with AN, the underlying mechanism is not yet known. Similar to those with ASD, it might be that attending to social stimuli results in hyperarousal, and the subsequent reduction in attention to the face represents an attempt to reduce arousal (Dalton et al. 2005; Hadjikhani et al. 2017). Indeed, behavioural evidence suggests higher sensitivity to social exclusion, as well as higher levels of social anxiety in AN (Cardi et al. 2018b; Kerr-Gaffney et al. 2018; Meneguzzo et al. 2020). Thus, attending to social stimuli or engaging in social interactions may be uncomfortable for some individuals with AN, resulting in avoidance and reduced overall looking times.

Another explanation is that reduced attention to faces in AN may be a result of reduced social motivation, a mechanism theorised to underly social deficits in ASD (Chevallier et al. 2012). Social motivation encompasses several psychological dispositions that bias humans to attend to social stimuli, seek and take pleasure from social interaction, and foster social bonds. Indeed, there is evidence to suggest these processes are altered in AN. Along with reduced attention to faces, Watson et al. (2010) showed that in a monetary choice task, individuals with AN did not sacrifice money to see faces as HCs did, suggesting possible alterations in the reward circuitry in AN. Further, individuals with AN show high levels of social anhedonia, with studies reporting scores similar to those reported in individuals with schizophrenia (Harrison et al. 2014; Tchanturia et al. 2012). Further research exploring the mechanism behind reduced social attention in AN may be helpful in understanding the nature of AN and ASD comorbidity.

A longer delay in orienting to the face was associated with higher levels of anxiety and depression (HADS scores) and functional impairment (WSAS scores) in the AN group only. Studies using eye-tracking in patients with major depressive disorder demonstrate increased looking times to sad faces and other negative information, compared to HCs (Keller et al. 2019). Further, there is some evidence to suggest reduced latency to first fixation on sad faces, a tendency which was associated with more severe depressive symptoms, in contrast to the association found in the current study (Duque and Vázquez 2015). Given the video clip in our study was not chosen for its emotional content (though actors displayed neutral or positive expressions), comparisons with the depression literature are difficult. However, paired with the lack of association in our REC and HC groups, the results suggest a unique relationship between depressive symptoms and social attention in AN. In relation to anxiety, attentional theories propose that anxiety is associated with reduced time to fixation on threat-relevant stimuli (indicating vigilance), differently to the direction of the relationship found in the current study (Weierich et al. 2008). In our regression analysis however, depression and anxiety did not explain a significant amount of the variance in time to first fixation. It could be that some other unmeasured factor, related to high levels of both anxiety and depression is responsible for the delay in fixation to faces.

Our findings have important clinical implications for understanding and treating individuals with AN. A wide literature has documented difficulties in various domains of social and emotional functioning in AN, including emotion recognition (Caglar-Nazali et al. 2014), theory of mind (Leppanen et al. 2018), and emotion regulation (Oldershaw et al. 2015). Despite this, social attention in AN has received very little consideration. Our findings, along with those of a recent study (Harrison et al. 2019) show that individuals with AN pay less attention to faces and core facial features when viewing dynamic, naturalistic stimuli, compared to HCs and REC. This may contribute to difficulties in social cognition, for example, in recognising emotions in others, as important non-verbal cues may be missed. In turn, these differences are likely to make social interactions and relating to others more difficult. In accordance with this hypothesis, greater work and social adjustment difficulties were significantly associated with a longer delay in attending to faces in the AN group only. Although difficulties in this domain were not associated with decreased overall looking times to faces and core features, this could be due to the measure used. The WSAS does not solely measure social functioning (it also assesses functioning in the home and private leisure activities) therefore our study may have benefitted from using a different measure, such as the Social Functioning Questionnaire (Tyrer et al. 2005) or the Friendship Questionnaire (Baron-Cohen and Wheelwright 2003), to further clarify relationships between social attention and self-reported social functioning.

Importantly, our results also suggest that differences in social attention in individuals with AN are due to ASD traits, rather than the eating disorder itself. Up to 50% of individuals with AN show high levels of ASD traits, scoring above clinical cut-offs on assessment tools such as the ADOS-2 (Westwood et al. 2018). Adaptations to conventional treatments for AN might be required for this group. For example, qualitative research has shown that individuals with AN and ASD report difficulties in communicating with one another and a lack of understanding of each other’s perspective, difficulties that are likely to interfere with the therapeutic relationship (Kinnaird et al. 2019, 2017). Providing psychoeducation to both patients and clinicians about different communicative styles and preferences may be helpful. Further, treatment modules designed to improve aspects of social cognition may be helpful for those with AN and high ASD traits. For example, social skills training groups have been shown to improve social cognition, friendship quality, and social skills knowledge in those with ASD (Hillier et al. 2007; Kandalaft et al. 2013; Laugeson et al. 2009; Schohl et al. 2014; Turner-Brown et al. 2008). Such interventions also report improvements in mental health outcomes, suggesting an association between social functioning and wider mental health (Hillier et al. 2011; Yoo et al. 2014). Whether such interventions might be useful for those with AN is a question for future research.

Although we cannot confirm whether participants in our study met full diagnostic criteria for ASD, the results have implications for our understanding of autism, particularly the female phenotype. Based on similarities in cognitive (e.g., set-shifting difficulties, weak central coherence, superior attention to detail, and theory of mind deficits) and behavioural profiles (e.g., perfectionism, rigid attitudes and behaviours, and narrow interests) some have argued that AN is a female manifestation of ASD (Gillberg and Råstam 1992). Indeed, in females with ASD, who are on average diagnosed later in life than males (Lai et al. 2015), a diagnosis often comes after many years of mental health service engagement for other conditions, including anxiety, depression, and AN (Lai et al. 2011; Mandy and Tchanturia 2015; Vagni et al. 2016). Several barriers to detection of ASD in females have been proposed. For example, diagnostic tools have been impacted by the longstanding gender bias towards male presentations, resulting in a lack of sensitivity to female presentations (Lai et al. 2015). On average, females with ASD are less likely to display repetitive behaviours and show better surface level social skills than males with ASD. Our findings suggest that reductions in social attention may be a transdiagnostic endophenotype, which may be helpful in understanding social cognitive processes beyond traditional diagnostic classifications. Future research examining non-social autistic symptoms in AN, such as repetitive behaviours or sensory sensitivities, may also help clarify the nature of ASD comorbidity in AN.

The current study has several limitations. Firstly, although our stimulus closely depicted what would typically be seen when interacting with others, the absence of real life interlocutor may have influenced eye movement patterns. A problem for much social attention research is that the tasks used are not inherently social—typically pictures or videos are presented on a computer screen. It may be that social attention differs in contexts where a real social interaction is expected, and this may be especially true in individuals with high levels of social anxiety, as in our study. On the other hand, our choice of a relatively complex stimulus may have introduced noise to the data. Although our aim was to provide a representation of what would normally be seen during a natural social interaction, variations in actor characteristics such as gender may have influenced attention allocation.

Another limitation concerns our measure of ASD traits. The SRS-2 has been shown to predict scores on clinical interview measures of ASD such as the ADOS-2 in individuals with AN (Kerr-Gaffney et al. 2020b), however it is possible that scores on the SRS-2 are influenced by symptoms of anxiety, depression, or alexithymia. Our study may have benefitted from including a sub-group of participants with AN and a diagnosis of ASD to further clarify our results. Relatedly, although our regression analysis showed that SRS-2 scores explained a significant amount of variance in time spent looking at faces, the final model only explained a relatively small amount of variance overall, suggesting other important factors influence social attention in our sample. While age, BMI, and psychiatric medication status were not found to be associated with social attention, it is possible that other demographic or clinical factors may influence attentional processes. The findings therefore require replication in other samples. Finally, although our results remained the same when males were excluded from analyses (see Supplementary File 2), it was not possible to examine whether attention differed between males and females with AN, due to the small proportion of males in our sample.

To conclude, our findings demonstrate the importance of comorbid psychopathology, and specifically ASD traits in social attention in individuals with AN. Past research has suggested considerable heterogeneity in social- and neuro-cognitive functioning in individuals with AN, possibly indicating differences in aetiological or maintenance factors (Renwick et al. 2015). Our results suggest that reduced social attention in AN may be a result of high ASD traits, in accordance with recent research demonstrating differences in set-shifting, emotion recognition, and empathic abilities in individuals with AN with and without high ASD traits (Kerr-Gaffney et al. 2020a, c; Westwood et al. 2017b). In order to clarify the mixed findings in related domains, future work examining social cognition in individuals with AN may benefit from including a measure of ASD traits, or indeed a sub-group of individuals with AN and a confirmed diagnosis of ASD.

## Electronic supplementary material

Below is the link to the electronic supplementary material.

Supplementary file1 (DOCX 27 kb)

Supplementary file2 (DOCX 24 kb)
