# Supplementary material for: Autistic Traits Mediate Reductions in Social Attention in Adults with Anorexia Nervosa
Source: J Autism Dev Disord. 2020 Sep 10;51(6):2077–90. doi: 10.1007/s10803-020-04686-y (PMC8124046; doi:10.1007/s10803-020-04686-y)
Supplement: Supplementary file 1 — Supplementary file1 (DOCX 27 kb) [file 10803_2020_4686_MOESM1_ESM.docx]

| Table 1. Correlations between attention, demographic variables, and psychopathology | | | | | | | | | |  |
| --- | --- | --- | --- | --- | --- | --- | --- | --- | --- | --- |
| *AN* | Age | BMI | EDE-Q | HADS-A | HADS-D | LSAS | TAS-20 | SRS-2 | WSAS | |
| AOI face | .01 | .22 | -.29 | -.21 | -.25 | -.20 | **-.33** | **-.40** | -.11 | |
| AOI eyes | .01 | .23 | -.22 | -.01 | -.20 | -.07 | -.14 | -.04 | -.18 | |
| AOI mouth | .12 | .04 | .09 | -.28 | -.07 | .04 | -.03 | -.25 | -.05 | |
| AOI nose | -.05 | .12 | -.09 | .13 | .13 | .03 | .01 | .03 | .03 | |
| Time to first fixation (face) | .07 | -.26 | .10 | **.34** | **.43** | .19 | .15 | .16 | **.37** | |
| Eye-to-mouth viewing ratio | -.14 | .08 | -.15 | .22 | .01 | -.07 | .00 | .22 | .01 | |
| *REC* | Age | BMI | EDE-Q | HADS-A | HADS-D | LSAS | TAS-20 | SRS-2 | WSAS | |
| AOI face | .01 | -.05 | .05 | .01 | .05 | .02 | -.03 | -.04 | .02 | |
| AOI eyes | .03 | .08 | -.16 | -.08 | -.11 | -.14 | -.14 | -.13 | .02 | |
| AOI mouth | .00 | -.20 | .03 | .08 | .11 | .07 | .13 | .14 | -.05 | |
| AOI nose | .02 | -.04 | .03 | -.05 | -.09 | -.05 | .06 | -.05 | -.10 | |
| Time to first fixation (face) | -.10 | .25 | -.07 | -.27 | -.18 | -.05 | -.07 | -.18 | -.26 | |
| Eye-to-mouth viewing ratio | .01 | .16 | -.08 | -.09 | -.13 | -.09 | -.14 | -.14 | .03 | |
| *HC* | Age | BMI | EDE-Q | HADS-A | HADS-D | LSAS | TAS-20 | SRS-2 | WSAS | |
| AOI face | .17 | .30 | .19 | .11 | -.11 | -.01 | -.12 | -.24 | .00 | |
| AOI eyes | -.06 | -.10 | .10 | -.16 | -.18 | -.22 | .00 | -.28 | .11 | |
| AOI mouth | .15 | .11 | -.08 | .26 | .14 | .19 | -.01 | .15 | .12 | |
| AOI nose | -.04 | -.02 | -.08 | -.03 | -.18 | -.15 | -.15 | -.16 | -.15 | |
| Time to first fixation (face) | -.06 | -.03 | -.08 | -.12 | -.05 | .01 | -.14 | -.15 | -.11 | |
| Eye-to-mouth viewing ratio | -.14 | -.08 | .15 | -.19 | -.19 | .17 | .01 | -.22 | -.15 | |
| AOI, area of interest; BMI, body mass index; EDE-Q, eating disorder examination questionnaire; HADS-A, hospital anxiety and depression scale, anxiety subscale; HADS-D, hospital anxiety and depression scale, depression subscale; LSAS, Liebowitz social anxiety scale; SRS-2, social responsiveness scale, 2^nd^ edition; TAS-20, twenty-item Toronto alexithymia scale  Significant correlations are in bold. | | | | | | | | | |  |
