# Supplementary material for: Autistic Traits Mediate Reductions in Social Attention in Adults with Anorexia Nervosa
Source: J Autism Dev Disord. 2020 Sep 10;51(6):2077–90. doi: 10.1007/s10803-020-04686-y (PMC8124046; doi:10.1007/s10803-020-04686-y)
Supplement: Supplementary file 2 — Supplementary file2 (DOCX 24 kb) [file 10803_2020_4686_MOESM2_ESM.docx]

**Analyses excluding males (n=7)**

**Attention to faces**

A one-way ANOVA indicated a significant difference in time spent looking at faces, F(2, 119) = 4.43, p = .014, ηp² = .07. Post-hoc analyses indicated that individuals with AN looked at faces significantly less (M=0.70, SD= 0.12) than REC (M=0.75, SD=.07), p=.045 (95% CI, -.09, -.01), and HCs (M=0.76, SD=0.07), p = .018 (95% CI, .01, .11)

Regarding time to first fixation, a one-way ANOVA indicated there were no significant differences between individuals with AN (M=0.82s, SD=0.52), REC (M=0.77s, SD=0.28s), or HCs (M=0.90s, SD=0.70s), F(2, 119) = 0.29, p = .75, ηp² = .01.

**Attention to facial features**

Mixed ANOVA with AOI as the within-subjects factor and group as the between subjects factor. A Greenhouse-Geisser correction was applied due to violation of Mauchly’s test of sphericity. The interaction between group and AOI was not significant, F(2.78, 165.52)= 0.73, p = .527, partial η^2^ = .01. The main effect of AOI showed a significant difference in the proportion of time spent looking at the different facial features, F(1.39, 165.52) = 11.91, p <.001, partial η^2^ =.09. Participants spent significantly more time looking at the eyes than the mouth (p=.025, 95% CI, .01, .11) and nose (p<.001, 95% CI, .05, .11). The main effect of group was also significant, F(2, 119) = 5.98, p = .003, partial η^2^ = .09. Post-hoc tests indicated that individuals with AN looked at AOIs less than REC (p=.013, 95% CI, -.03, -.01) and HCs (p = .006, 95% CI, .01, .04).

A one-way ANOVA indicated there were no significant differences in eye-to-mouth viewing ratio between individuals with AN (M=0.59, SD=0.26), REC (M=0.61, SD=0.25), and HC (M=0.53, SD=0.27), F(2, 119)= 0.81, p=.448, ηp² = .01.

**Relationships between social attention and psychopathology**

In individuals with AN, time spent looking at faces was significantly negatively correlated with TAS-20 (r=-.34, p=.041) and SRS-2 scores (r=-.41, p=.011), while time to first fixation to the face was significantly positively correlated with WSAS (r=.41, p=.012), depression (r=.42, p=.010), and anxiety scores (r=.38, p=.019). No significant correlations were found in HC or REC groups.

Hierarchical regression (Dependent Variable: time spent on faces): The final model (model 3) was significant, R^2^=.10, F(4, 118)=3.06, p=.019, adjusted R^2^=.07. The addition of SRS-2 scores led to a significant increase in R^2^ (model 3), however the addition of TAS-20 scores did not (model 2). In the final model, only SRS-2 scores made a significant unique contribution to explaining the variance in time spent looking at faces (see Table S1)

| Table S1. Hierarchical regression analysis predicting time spent looking at faces from associated psychopathology scores | | | |
| --- | --- | --- | --- |
|  | Model 1 | Model 2 | Model 3 |
| Group |  |  |  |
| AN vs HC | -.26* | -.20 | -.15 |
| REC vs HC | -.04 | .00 | .06 |
| TAS-20 |  | -.11 | .07 |
| SRS-2 |  |  | -.27* |
| R^2^ | .06 | .07 | .10 |
| Note: Figures shown are standardized coefficients. Group was represented as two dummy variables.  * *p* <.05 | | | |

Mediation: Bias-corrected bootstrapped CIs for the indirect effects were entirely below zero (*b_1_* = -.03 [-.05 -.01], *b*_2_ = .02 [-.04 -.01]), indicating there was a significant mediation effect of group on time spent looking at faces through SRS-2 scores. The direct effect of group was not significant (*c*_1_ = -.03, *c*_2_ = .01, *p* = .104), indicating that group did not influence time spent looking at faces independent of its effect on SRS-2 scores.

Hierarchical regression (Dependent Variable: time to first fixation): The full model was not significant, R^2^=.02, F(4,117)=0.70, p=.595, adjusted R^2^ = -.01. None of the included variables explained significant variance in time to first fixation on the face (see table S2).

| Table S2. Hierarchical regression analysis predicting time to first fixation on the face from associated psychopathology scores | | | |
| --- | --- | --- | --- |
|  | Model 1 | Model 2 | Model 3 |
| Group |  |  |  |
| AN vs HC | -.05 | -.03 | -.11 |
| REC vs HC | -.08 | -.06 | -.07 |
| HADS-A |  | -.04 | -.17 |
| HADS-D |  |  | .22 |
| R^2^ | .01 | .01 | .02 |
| Note: Figures shown are standardized coefficients. Group was represented as two dummy variables.  * *p* <.05 | | | |
